# Qualitative Analysis of Student Reflections on Preclinical Dental Implant Education

**DOI:** 10.3390/dj12090293

**Published:** 2024-09-13

**Authors:** Hassan Ziada, Michael Webberson, Rassilee Sharma, Neamat Hassan Abubakr

**Affiliations:** 1 Clinical Science Department, School of Dental Medicine, University of Nevada, 1001 Shadow Lane, Las Vegas, NV 89106, USA; michael.webberson@unlv.edu (M.W.); rassilee.sharma@unlv.edu (R.S.); 2 Biomedical Sciences Department, School of Dental Medicine, University of Nevada, Las Vegas, NV 89106, USA; neamat.hassan@unlv.edu

**Keywords:** reflections, dental implants, preclinical, qualitative design

## Abstract

Dental implant education is required to prepare students for independent general practice. This investigation aimed to assess students’ perceptions of their educational experience and training in a preclinical dental implant introduction course, using reflective logs anonymously extracted from course portfolios. Methods: This study employed qualitative research methodology to analyze second-year dental students’ reflections on their educational and development of psychomotor skills in a preclinical course focusing on dental implants at the University of Nevada, Las Vegas. These reflections served as the primary data source for qualitative analysis. The analysis was facilitated using NVivo software version 12 plus, which assisted in data coding and the organization of these codes into meaningful units, patterns, and themes. Results: Four themes emerged, which interrelated to each other and to the research question. Students reported positive course outcomes in dental implant learning, improvements in applying theoretical implant knowledge while developing practical skills, digitally scanning implant cases for the final restoration, and enhancement of their insight in evidence-based restoratively driven implant planning. They generally found the hands-on experience to have improved their understanding of the dental implant as an option for restoration. Although there were challenges, students viewed these as learning opportunities. For us, as educators, it provided invaluable feedback to understand students’ perceptions of difficulties in knowledge acquisition and psychomotor skill development in placing and restoring dental implants. Conclusions: Within the limitation of this study, students expressed a positive perception of their learning experience in the introductory course on dental implants.

## 1. Introduction

Recent trends in dental education have seen a shift towards incorporating predoctoral dental implant education, a departure from its traditional postgraduate focus [1]. This shift is in response to the growing consensus that competency in dental implant procedures is essential for dental graduates preparing for independent general practice [2,3]. The European consensus for undergraduate dental education indicated the need for implant dentistry education into the dental curriculum [3]. As a result, implant dentistry has been progressively included in European undergraduate dental education. The Commission on Dental Accreditation (CODA) standard in the United States for predoctoral programs also mandates that dental graduates be competent in the “replacement of missing teeth”, including the use of dental implants [4]. The implementation of implant dental education has also been accelerated as a response to align professional training with the growing treatment needs of populations [5]. Consequently, dental educational institutes globally are expanding and continuously enhancing their implant education programs [6].

To reach the desired competencies, the undergraduate/preclinical pedagogy should include understanding the fundamentals of osseointegration, the related biomechanical principles, and prosthetically driven treatment planning [7]. 

In 2009, Hicklin et al. reported more specific pedagogical requirements, which included detailed characteristics of etiology, clinical and diagnostic parameters, treatment planning, esthetic and soft tissue considerations, implant site development and modifications, osseointegration, implant materials, implant surgery and its related complications, differences between fixed dental prosthesis against implant reconstruction, type of restorations, biomechanics, evidence-based long-term outcomes, implant management, the pathogenesis of implant-related inflammation prevention, and the management of peri-implantitis [8]. These requirements should allow dental students to be able to discuss the risks and benefits analysis of implant therapy and the biological and financial cost of implants in an overall treatment plan. They should also be able to describe the indications and contraindications of implant placement and the types and techniques of implant restorations [9,10]. Achieving these outcomes requires a well-designed preclinical course with close supervision accompanied by reflective learning emphasizing evidence-based practices, which is central to implant therapy, education, and application.

Student reflections on dental implant education are invaluable for curriculum development and refinement of implant courses [11]. The growing emphasis in healthcare education on reflective practice, often documented through student portfolios, aligns with this thought [12,13]. Reflective practice fosters deep thinking, critical appraisal, and an open-minded approach to problem-solving, thereby enhancing professional confidence [14]. In dental education, it fosters a dynamic patient–dentist relationship and encourages students to engage in intellectual reflection [15]. However, not all students may naturally engage in reflective practice, and practical skill development might take precedence over reflective learning [16,17]. Nevertheless, reflective practice is important and should be reinforced through course design and repetitive exercises [18]. A ticking box process for reflections may make it difficult to conduct an in-depth analysis since reflections are qualitative in nature and, accordingly, more suitable for analysis through qualitative methodologies, as in the current study [19,20]. Furthermore, self-assessment is anchored in reflective activities, and if both are developed early in a preclinical setting and strengthened later in the clinical environment, it will help students become more independent reflective clinicians [5]. In dental education, in particular, it should be encouraged and reinforced since it helps students honestly and proficiently self-assess [6]. Therefore, reflective logs similar to those used in this study should allow educators to understand students’ voices, insights into their learning experiences, and perspectives [21]. It also improves students’ reflective ability and allows them to rationalize mistakes and clarify contradictions. It also reduces students’ hesitation and uncertainty and eliminates any possible lack of self-confidence [21].

This reflective discourse enhances students’ reflective abilities and also enables them to understand and rationalize their learning experiences. Furthermore, it raises confidence and reduces hesitation in decision-making [21]. Furthermore, according to Kolb’s “experiential learning cycle”, reflective experiences in one discipline can provide insights and steps to take for future encounters in the same discipline or other disciplines [22]. Reflection is student-centered, as it allows them to link their reflections to their learning and skill development, which, if investigated and analyzed, should influence teaching philosophies and reshape learning [23]. In addition, students’ growth is thought to be related to their engagement in reflecting on their learning process [24]. As such, the use of reflective documented dialogue engages students in the process of self-appraisal, which helps them identify their own learning needs, a self-directed learning process, and the ultimate goal that underpins the concept of lifelong learning, particularly in implant dentistry, where dentists are required to engage in continuous professional development [25].

### Conceptual Framework

**Figure 1:**
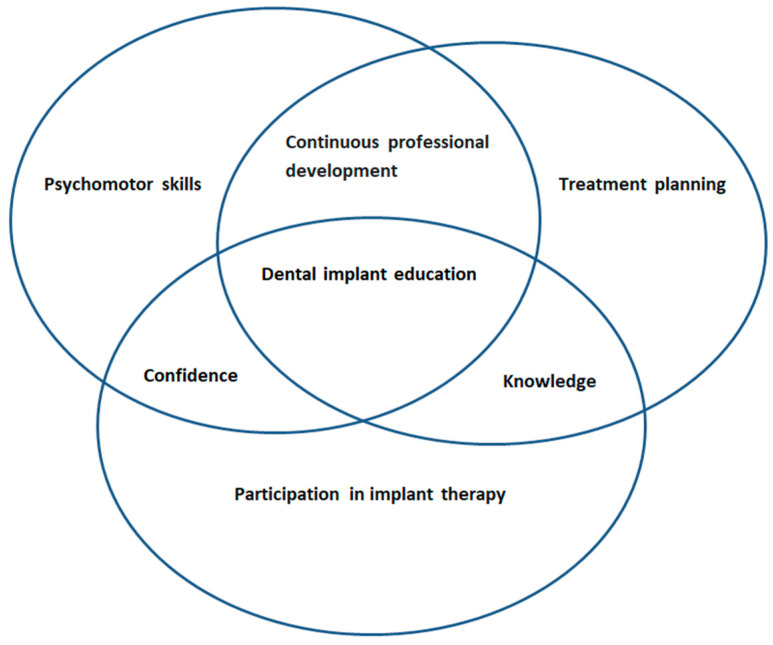
An introduction to dental implants in dental education illustrated with a conceptual framework.

Figure 1 demonstrates the literature on dental implants in dental education. It is depicted in a multilayered context in overlapping circles representing the interrelationship between the influences on preclinical dental implant education. The research question in this study is as follows: what are the preclinical students’ dental implant education experiences and their reflections on their progress and development? This study aims to evaluate students’ perceptions of their education and psychomotor skill development in a preclinical course on dental implants, utilizing reflective dialogue as a key tool.

## 2. Materials and Methods

The present study used a qualitative research approach to analyze the reflections of students’ educational experiences in an introductory preclinical course on dental implantology. Second-year dental students were the target group, and their reflective logs were the data for the qualitative analysis [26]. The use of reflective logs as a data source has been accepted in qualitative research as a data source, which can also originate from various sources, including narrative documentation [27].

The contextualization of the study and reflection collection did not occur until after the completion of the course, adhering to ethical standards of educational research where the students had to advance to the subsequent academic year before the start of the study. Hence, this study is a qualitative retrospective study. Prior to analysis, the reflective logs were anonymized and de-identified. This study received an “Exempt” status from the Office of Research Integrity—Human Subjects at the University of Nevada, Las Vegas (UNLV-2022-643). The inclusion criteria were de-identified and anonymized reflective logs for the practical part of the course DEN7226L. These logs were then scanned, assigned arbitrary numerical identifiers, and filed for analysis by a staff member not involved in this study. 

**Figure 2:**
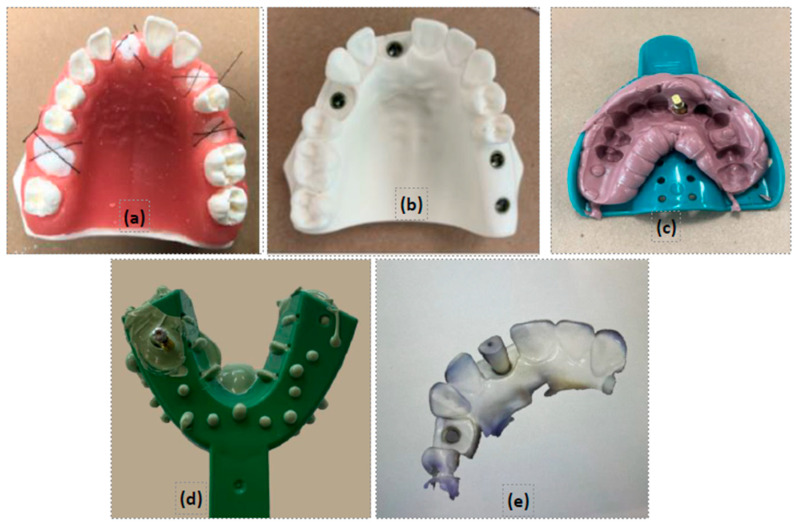
Images taken by students from reflection logs. (**a**) socket preservation, (**b**) implant placement, (**c**) closed tray impression, (**d**) open tray impression, and (**e**) digital implant impression.

During the summer term of the 2021/2022 academic year, all 89 students from the second-year dental class (DS 2) participated in the courses DEN 7226 (didactic), titled “Introduction to Dental Implants” and its practical component as DEN 7226L (laboratory). These 14-week courses were designed to teach theoretical knowledge on dental implants, prosthetically driven treatment planning, and also hands-on practical applications on mannequins. Students were required to create a detailed portfolio as part of their evaluation, recording their clinical simulated activities throughout the semester. This portfolio included photographic records of all practical exercises, such as socket preservation techniques utilizing Geistlich^®^ products, implant placements on typodont models (ACT and Paradigm), and both conventional and digital implant impressions (Figure 2). Additionally, the portfolio incorporated self-evaluations and faculty evaluations based on specific rubrics and a reflective log. This reflection-on-action log reflected the detailed students’ progression and skill development throughout the course and contributed 10% to their final grade.

### 2.1. The Simulation Activity under Investigation

At the beginning of the semester, during the course orientation, students were instructed in the principles of reflective practice. They were also provided with a model portfolio to serve as a guide. They were asked to document reflective dialogues (reflection-on-action) of their progress and development, including challenges and difficulties encountered and their approach to overcoming these.

Following best practices for qualitative research, the students’ written reflections were initially carefully read multiple times. This process is usually aimed at obtaining a general understanding of the students’ perceptions before coding, understanding the data, and identifying prominent features and patterns. Thereafter, coding steps were made to closely evaluate the data and detect prominent features and patterns. The written text data were segmented into separate units based on their meanings, events, or ideas. These units were subsequently analyzed to identify their similarities and differences. Coding was then independently made and followed by interpretive descriptions and categorization into more abstract groupings. This process was completed iteratively to ensure that the analyses remained consistent with the data presented.

### 2.2. Thematic Analysis

Each author independently performed the coding task using a code book (Table 1). Subsequently, a comparative analysis of the codings was undertaken to identify commonalities, parallels, and discrepancies. A series of revisions formed the validation process. This process facilitated the refinement and consolidation of the codes, enabling the selection of themes through a methodological process of “cutting and pasting” from the codes to the themes which allowed for the development of association, correlation, and amalgamation of themes [28]. The next phase involved a thorough examination of the scripts under each code, for their relevance and association with the research question. A further profound understanding of the reflections and their integrity was achieved through these iterative readings and the collaborative discussions among the authors. This rigorous approach ensured that the analysis was thorough and coherent with relevant relationships and interconnections [26]. Theoretical saturation was reached by the authors’ agreement after investigating the reflections of the 89 participants.

Thematic analysis was then conducted in an iterative and inductive way, focusing on describing and interpreting the chosen codes [29]. The Braun and Clark 2006 qualitative descriptive independent six-stage approach was used, where the emerging themes describe narratives relevant to the research question. This staged approach has demonstrated its effectiveness in research within healthcare and education [30]. The use of NVivo software version 12 plus (QSR International Pty Ltd., Vic, Australia) was instrumental in facilitating the coding process. It improved the organization of the data into entities, which were then systematically arranged into patterns and themes. The software also maintained the ongoing review and analysis of the data, as well as the integration of new codes as deemed necessary.

### 2.3. Reliability

To maintain the credibility of the qualitative analysis, the researchers engaged independently in the coding and categorizing of the data (author triangulation). In addition, the preliminary findings of the analysis underwent thorough and repeated reviews. We also used the known efficient analysis of an inductive and deductive approach, with several repetitions and fine-tuning and of developing a codebook (Table 1) essential in validating rigor [31,32].

## 3. Results

The thematic analysis organized the codes into broader themes, making understanding and reporting the core aspects of students’ reflections easier. Based on the initial analysis, four prominent themes have emerged as the most significant and frequent within the dataset. These themes offer a robust basis for further in-depth exploration and discussion. With four themes, we can address various perspectives or components relevant to the research question. This approach ensures understanding and provides depth of interpretation of the complex students’ experiences.

The conventional approach to presenting qualitative data involves using direct quotes from participants, italicized, to demonstrate themes. These are as follows:

Dental implant learning

Potential improvements

Digital trends and further implant education

Treatment planning and evidence-based practice

### 3.1. Theme 1: Dental Implant Learning

Many participants mentioned having learned much from the course and exercises, suggesting that learning effectively delivered comprehensive knowledge.

*“I felt like I learned a lot from this course due to the fact that we were able to implement and apply what we learned from the PowerPoint into the projects that we did”*.

(Participant 29)

Regarding the skill component development, a significant number of participants reported acquiring hands-on skills, like suturing techniques and socket preservation, suggesting that the solid practical component allowed students to gain hands-on experience.

*“I learned how to place the bone grafting material, collagen matrix disc, and how to secure it all with a figure eight suture”*.

(Participant 2)

*Learning how to suture was also very useful because I was able to implement it in clinic*.

(Participant 62)

*“My suturing abilities improved tremendously through the socket preservation lab”*.

(Participant 57)

Given the meticulous nature of implant-related procedures, the learning experience increased students’ appreciation of the importance of attention to detail in learning.

*“I learned the importance of paying attention to small details and measuring multiple times prior to proceeding with the procedure to ensure the best results and longest survival rates of implants”*.

(Participant 65)

The student narrated how the learning opportunity improved their theoretical knowledge and practical experience, which appears to have enhanced both comprehension and skill acquisition and bridged the gap between theoretical and practical skills.

*“Attending this Implant course was really a great learning opportunity for me to improve both my theoretical as well as practical skills”*.

(Participant 43)

*“I feel that overall I have improved in my knowledge and skill of placing implants, although I know that this is a very basic introduction and there are many more years of experience to be had”*.

(Participant 11)

Some participants indicated that they enjoyed the experience and were looking forward to eventually applying their skills to their patients, suggesting the course instilled confidence and enthusiasm that they can indeed, at this early stage, be more comfortable discussing the process with their patients.

*“I know I’m not ready to place and restore implants from start to finish, but I certainly feel much more comfortable discussing the process with patients*.

(Participant 17)

*Above all else, the hands-on learning made the experience not only engaging but also memorable”*.

(Participant 56)

The experience expanded students’ understanding beyond implant therapy, learning more about patient management, treatment planning, and holistic dental care.

*“I am very glad that I had the chance to attend implant course which has expand my knowledge to a level where I can feel confident treating patient”*.

(Participant 44)

This student also reflected on their personal experience in the context of their learning.

*“I was really intrigued in taking implants since my mom is currently in the process of receiving an implant. I was confused as to why her dentist was waiting so long to place the implant when bone loss is such a risk, but I came to find out that he was actually doing socket preservation on my mom”*.

(Participants 19)

As expected, there were more reflections on the practical aspects of learning, which seemed to bring enjoyment and reflections on strengths and weaknesses to student learning skill development.

*“I really enjoyed being able to get hands-on experience with implant placement, socket preservation, scanning, and open/close tray impressions. Also, being able to have these activities done in class gave me more of an idea of what my strengths and weaknesses are when it comes to implant related lab work”*.

(Participant 23)

However, some students felt that they should have had more experience by including more practical assignments.

*“I could use more practice in placing implants and scanning digitally”*.

(Participants 19)

The experience seems to have instilled confidence in students. This confidence came with a responsible attitude and realistic expectations.

*“Even though I feel confident and comfortable in my abilities, I have a long way to go to master the art of implants”*.

(Participant 4)

As course directors, we can comfortably say that the experience in this introduction to dental implants was efficient in preparing students for real-world challenges.

*“Although we have not done so on a living patient, this class has exposed us to enough scenarios that we should feel comfortable working on an implant cases in the future”*.

(Participant 82)

Overall, the learning was a positive experience. Participants seemed to benefit from the blend of theory and practical exercises, enhancing their skills and understanding of the use of dental implants in dentistry.

### 3.2. Theme 2: Potential Improvements

The areas of improvement included specific areas of the learned skills.

*“Some areas of improvement include trying not to place the grafting material and collagen matrix disc is way below the crest of the bone. The areas I would need more practice in is packing the membrane and executing the figure eight suture”*.

(Participant 2)

Another potential improvement was in this narrative, reflecting on the quality of work and speed.

*“I need to work on the time it takes me to suture as well as my quality because I don’t want to give patients a bad suture that will also heal badly”*.

(Participant 4)

In this regard, the concept of socket preservation with its related suturing was a challenge, and although the students enjoyed and appreciated it, the learning curve and the potential improvements were discussed.

*“After some more practice, I was able to successfully place the graft material and suture”*.

(Participant 40)

The progress towards improved skills was also reflected on.

*Prior to this exercise, I had not sutured before. This exercise was good practice for me to practice suturing. From this experience, I was able to place better sutures*.

(Participant 41)

The main goal of the simulation course is to prepare students for a restoratively driven treatment planning concept; achieving this goal was evident.

*“I know I’m not ready to place and restore implants from start to finish, but I certainly feel much more comfortable discussing the process with patients and working on a case”*.

(Participant 17)

The development of psychomotor skills and the related confidence levels were understandably variable among students. This was reflected on, in this reflection.

*“After my 4th try on the typodont, I improved my hand-skill and was more precise in my placement”*.

(Participant 21)

*“My suturing abilities improved tremendously through the socket preservation lab”*.

(Participant 57)

Reflecting on impression making, for both the conventional and digital formats, and how to improve on this skill in these courses were also discussed.

*“For the open tray, I again had a void on the distal side, meaning I did not surround the entire implant coping carefully with PVS before placing the tray. I will also need to work on minimizing air bubbles with the PVS. In the digital impression, An area I could improve on. I was being more steady and thorough with my scanning because I felt like I had to scan over the same area multiple times since I could not get the angulation perfect”*.

(Participant 26)

The challenges faced included any common errors made, or difficulty in understanding certain concepts, and the reflections on these during the course are helpful in improving psychomotor skills.

*“I struggled with the hand coordination for the first few times but ended up picking it up on the last try”*.

(Participant 4)

The challenges with specific parts of the hands-on experience and how these were overcome were evident in these reflections.

*“I was confused in the beginning with the many different parts of the implant placement and impression process, but the multiple exposures helped me to become more familiar and comfortable with the pieces”*.

(Participant 84)

Some narrated how confusions were clarified once the hands-on exercises occurred.

*“I felt a little confused about some of the concepts like open and close trays, but when we did them in lab it started to make a lot more sense”*.

(Participant 29)

One of the most important goals of this course is for the students to recognize the implant-related components. This is to aid students in identifying and ordering the correct component when students are in the clinic. This proved challenging and more effort from teachers must be implemented to overcome this issue.

*“Initially I had trouble recognizing which components to use in open and close tray techniques”*.

(Participant 44)

Understandably, some of the students found the open-tray impression techniques more challenging. Perhaps the need for several steps in impression making and removing the tray might have resulted in feeling that way.

*“Open tray impressions were harder than close tray, and I need to make sure that the hole around is big enough”*.

(Participant 49)

However, for some, both conventional impression techniques were challenging.

*“To my surprise, closed and open tray impressions were challenging to capture proper margins and required technique-sensitive awareness”*.

(Participant 58)

Students’ reflections also revealed general feedback about the course content and delivery. For example, the surgical guide was 3D printed and ready for the student. Some students expressed interest in being involved in the process of construction of a 3D-printed surgical template. Also, there was a desire to go through all steps and all the way to the final step of crown construction and cementation/screw retention.

*“I do wish we could have made our own surgical guides. It also would have been nice to fabricate some implant crowns”*.

(Participant 3)

There was also an interest in completing the implant installation on pigs’ jaws rather than typodont models to obtain a closer-to-reality installation.

*“I also think in the future if we could possibly place implants in an actual pig jag could truly improve the course”*.

(Participant 77)

The new terminology in this course was challenging to some students, which we will give more consideration to in the future.

*“The terminology was the biggest mountain to overcome in the course. Instructions would be given with words like analog, pick up/open tray technique, transfer/closed tray technique, abutment, implant body, locators, torque wrench, driver, etc., and it was very difficult to follow”*.

(Participant 54)

The students also reflected on what they found as their favorite sessions in this course, which was variable. Understanding what participants enjoyed the most can shed light on what is working and what should be retained or enhanced in the curriculum.

*“My favorite lab session was socket preservation. It taught me how I can preserve the bone volume and prevent bone loss by treating the socket with collagen matrix and muco-graft seal”*.

(Participant 5)

*“My favorite lab session was using the scan body and implementing the digital scanners on the implants”*.

(Participant 12)

*“My favorite part of this course was initially learning how to place the implants using the surgical stent and verifying the placement with radiographs”*.

(Participant 68)

However, there was also the expression of finding the implant installation sessions as the least favorite. Although implant placement is not a requirement for graduation at this school, it was intended to enhance psychomotor skills, and it was also implemented because it helps in the conceptualization of treatment planning.

*“My least favorite exercise was implant placement because I think it had very little room for error and it was the most stressful to get correct on the first try”*.

(Participant 26)

### 3.3. Theme 3: Digital Trends and Further Implant Education

The experience of scanning and designing implants was perceived as informative, recognizing the growing importance of digital dentistry.

*“Scanning and designing the implant was informative”*.

(Participant 15)

As the field advances in digital dentistry in implantology, it is essential to know if participants feel equipped with the latest digital dentistry techniques and their applications.

*“The digital impression lab session was also very helpful to see how implant impressions and designing can be done digitally”*.

(Participant 5)

Furthermore, the digital trend is moving at a rapid pace, and students feel that it is important to learn more and become skilled in digital implantology.

*“I believe it is crucial for us as students to understand the uses of the digital hardware and their supporting software”*.

(Participant 7)

Regarding the use and accuracy of digital technology, they found it easier to take a digital impression than a conventional one., however, they recognized THAT this was a bench experience in a simulation, which is different compared to real-life scenarios on an actual patient.

*“Digital Impression: This was probably the easiest impression since we just had to place the scanning body and scan it but I think it would take more technique if it were in an actual patient”*.

(Participant 30)

Some reflected on their technical skill in digital technology and how they improved and on finding ways to improve these skills.

*“Initially scanning the occlusal surfaces of the teeth was not difficult. However, I was having difficulty trying to capture the interproximal areas of the teeth. One aspect that I noticed to improve on was being able to adapt the scanner at an adequate distance and angle onto the teeth”*.

(Participant 42)

Notwithstanding, the challenges of a new learning experience were narrated.

*“Digital Impression were a little more challenging than I thought, but again, more experience will help me improve my technique”*.

(Participant 21)

For the educators moving forward, we were also alerted that another more advanced course may be needed as there is a strong student interest in further development.

*“I need more practice with digital scanning”*.

(Participants 19)

*“I believe with additional practice, future courses and experience, I will feel much more confident”*.

(Participant 21)

For the students, it is evident that the course instilled the concept of further education, development, and life-long learning.

*“I think the most important takeaway for me was that it is going to take continual practice to get more comfortable with the execution of the implant process”*.

(Participant 45)

In general, students were very grateful for learning the option of digital planning and impression making in this early introduction to dental implants. Participants also acknowledged the benefits of digital technology in dentistry, particularly in impression scanning, treatment planning, and creating accurate 3D digital surgical stents.

*“I’m especially grateful to have got some experience with digital scanning since that’s going to be the future of the industry”*.

(Participant 81)

### 3.4. Theme 4: Treatment Planning and Evidence-Based Practice

One of the aims of this course was to encourage students to appreciate the importance of restoratively driven dental implant planning. This aim seems to have been achieved.

*“What I found most helpful about this class was not placing the implants themselves, but the patient selection process beforehand and the restoration procedures after placement. Careful planning and having the finished product in mind will aid in better outcomes”*.

(Participant 3)

Also, another student stated:

*“I appreciated how an emphasis was placed on restorative-based treatment planning”*.

(Participant 39)

They also progressively saw how the planning concept for dental implants can be incorporated into the overall treatment planning.

*“As for my personal progress, I entered 7226 with an adequate understanding of implant basics, but as the lectures and assignments progresses, I feel like I have a clinic-ready understanding of when and how to implement them in treatment planning”*.

(Participant 9)

Bridging the gap between the simulated environment and that of the clinic, particularly related to treatment planning, makes students feel comfortable that the gap has been narrowed or eliminated.

*“I feel much more confident in discussing implant related treatment with patients and other dental providers”*.

(Participant 32)

*“When I begin planning implant procedures for my patients in the future, I now believe I have the tools to give them the most realistic expectation of their implants in terms of what to expect from the procedures, the procedural timeline, and how implants will function with the rest of their dentition”*.

(Participant 63)

There was also a sense of self-satisfaction and achievement in reaching the main goal of the ability of treatment planning.

*“I have fulfilled my goal of being able to identify what to look for when planning an implant and how to plan the steps correctly”*.

(Participant 34)

Also, another student added:

*“As the semester progressed, I became more comfortable with the course assignments and with the overall procedures and treatment planning associated with placing implants”*.

(Participant 55)

Furthermore, there was also the appreciation that treatment planning for implants is a complex process that requires careful attention to detail.

*“This class helped me to appreciate the complexities and subtleties of the implant process. I now have a greater understanding of the steps that an implant takes, how long the process to complete one is, and what criteria make a patient a good candidate for implants”*.

(Participant 78)

Although using surgical guides during implant placement was a new concept to students, they immediately felt comfortable using them and could see the benefits of their use.

*“I saw the value of a surgical guide and how critical it would be to correctly placing an implant”*.

(Participant 6)

Students also reflected on the technical insight in the development of their skills and clinical judgment.

*“I was using it as a definitive spot for #14 and 15, however I did course correct and aligned my drilling for #5 and 8”*.

(Participant 11)

Students acknowledged the need for careful implant placement steps even in the presence of a well-designed surgical template.

*“This exercise allowed me to realize how easy it is to be out of alignment despite having a surgical guide”*.

(Participant 41)

*“I discovered that having a guide is often not enough to place an implant on the right position”*.

(Participant 52)

Finally, there was insight and appreciation of the efficacy of case-based learning and assessments and evidence-based learning in the PICO exercises.

*“The integration of PICO was also extremely helpful in facilitating my learning as I was able to answer many of my own questions”*.

(Participant 23)

## 4. Discussion

The present investigation sought to analyze the reflections of students in a preclinical course on dental implantology, focusing on their retrospective cognitive activities, “reflection-on-action”. Future research should consider a simultaneous examination of both “reflection-in-action” and “reflection-on-action” [15].

Reflective practices in educational settings are inherently student-centered and can enhance our understanding of the progression and challenges in dental students’ competency challenges. Such self-assessment is not only beneficial for patient care but also fosters a culture of lifelong learning. Encouraging students to independently reflect and evaluate their competency development offers insights into their cognitive processes, understanding, rationalizing, and evaluating their approaches to situations encountered during their skill acquisition. Consequently, actively asking for students’ reflective dialogues in psychomotor skill development is a justified and valuable educational strategy [33]. It helps students learn about themselves, and it should also help us, as educators, to improve our teaching strategies [34]. Furthermore, reflection in education strengthens the teacher–student relationship, thereby enhancing the learning experience and preparing students for real-world dental practice [35,36]. Recent studies indicated the importance and significance of introducing dental implantology education at an early stage, with a particular emphasis on preclinical training [37,38].

This course ignited a passion for further study in implantology, narrated repeatedly by participants. This can be a measure of success that the course fostered a more profound interest in the field. This finding echoes the positive feedback and increased motivation for implant therapy in the future, reported by Schweyen et al. (2020) [6]. Similar studies have also indicated that early exposure to implant dentistry during dental education encourages greater involvement in implant practice post-graduation [39,40]. In addition, Kido et al. (2009) found predoctoral implant education to have deepened students’ understanding of implant treatment [39] and increased students’ confidence and satisfaction levels [41,42]. In a similar qualitative study, Afshari et al. 2014, found that predoctoral dental students benefited from their educational experience and would likely increase their knowledge and skills through formal education after graduation [43]. However, the integration of dental implant courses in predoctoral education faces challenges, including funding constraints and curricular time limitations [1].

Some students experienced difficulties in reflecting on their experiences, possibly due to their limited prior experiences [44], a finding also reported by Wong et al. [45]. These students tended to describe their experiences rather than engage in reflective thinking. Also, the grade of only 10% for a portfolio that contains the reflection might have resulted in less incentive to provide more in-depth reflections. Of course, there may also be a variance in students’ inclination or ability to reflect. Additionally, the primary focus of predoctoral students on skill acquisition and development might overshadow the perceived importance of reflective practice. Consequently, these students might not fully benefit from reflective learning in their future endeavors [26] and may view reflection as an extraneous or non-essential component of their education [46]. Certainly, reflective practice is essential for any graduating dentist, with the literature associating it with improved academic performance [47]. This study’s limitation is that it only included qualitative data. Hence, future studies should consider a mixed-method analysis that might produce more robust findings regarding students’ self-reflection on their implant skill development. Another limitation is that written reflections might be an experience some students consider uncomfortable. Furthermore, some students may not consider the concept of reflection a “real” study [46].

## 5. Conclusions

The findings indicate that students perceived their introductory experience in dental implantology positively, particularly appreciating the incorporation of hands-on practical training and the inclusion of a digital implant impression process. This exposure not only improved their confidence but also their perceived ability to effectively apply the acquired knowledge and skills.

This qualitative study provided information that can help open more comprehensive investigations into the learning processes of students in the context of implant dentistry. Future research should aim to deeply evaluate the details of knowledge acquisition and skill development in this field.

## Figures and Tables

**Table 1:** Code book.

Code Label	Characterization	Account Description	Criterion	Example
Learning experience	The learning through the semester	Perception of learning	Can be better learning or no learning	This class allowed me to learn all the different steps required to place dental implants (Particpant 8).
Challenges and mistakes	Challenging knowledge or skill acquisition	Efficiency in understanding and undertaking tasks	Can be challenging or not or initially challenging and later became neutral	By far the most challenging of exercises was the socket preservation technique, specifically the defective socket. Not only was the suturing difficult, but placing the artificial membrane that would prevent the invasion of epithelial cells in a live patient was very technique sensitive (Participant 83).
Hands on experience	The impact of simulated hands on activities	How helpful or not the hands on exercises on psychomotor skill development	Helpful in skill development or neutral	It was very helpful to have some hands-on experience to supplement the didactic portion of the course as I can relate more with hands-on activities than I typically would with just didactic/ppt presentations Also, being able to have these activities done in class gave me more of an idea of what my strengths and weaknesses are when it comes to implant related lab work. Thus, giving me an idea of what I should practice before taking on an implant case (Participant 23).
Areas of Improvements	Initially struggled but eventually improved	Students perception of their struggles	The room for improvement in skill	The impressions were moderately difficult with the open tray being more difficult than the closed tray. However I see now how to get a good open tray impression, although my first one was not ideal and took multiple attempts (Participant 71).
The course	The effectiveness of learning and the expectation	The perception of how course facilitate learning	Expectation may or not be fulfilled	This course amply fulfilled all my expectation and beyond. I did not just learned how to place implants but also learning how to restore them and actual being able to take analog impressions with both techniques open and close tray plus learning it the digital way with a scan body (Participant 50).
Practical activities	The favourite exercises in psychomotor skill development	Students‘ perceptions of how much the found one activity or more enjoyable	The experience can be enjoyable or not enjoyable	The socket preservation exercise was also an experience I enjoyed and probably something I will do a lot more of as a general dentist (Participant 21).
Confidence	Acknowledged of increase in confidence	Students’ voice of their confidence levels	The ability to apply knowledge to real-life scenarios,	In regard to implants, I feel much more confident in discussing implant related treatment with patients and other dental providers (Participant 32).
Interest in further implant education	Given the knowledge and experience, how students perceive the need for future implant education	Student’s perception of the need for further dental implant education	The need for more practice to build on the learned techniques	I believe with additional practice, future courses and experience, I will feel much more confident on placing implant at the right location and angle (Participant 21).
Digital dentistry in implantalogy	The opportunity to learn and practice digital implant dentistry	Students perception of using digital technology in implant dentistry	The learning opportunity can be positive or negative	As the field of dentistry is moving towards the digital trend at a rapid pace, I believe it is crucial for us as students to understand the uses of the digital hardware and their supporting software (Participant 7).
Treatment Planning in implant dentistry	The importance of treatment planning	Students’ perception the importance of treatment planning	The treatment planning ability has or has not been fullfield	I entered 7226 with an adequate understanding of implant basics, but as the lectures and assignments progresses, I feel like I have a clinic-ready understanding of when and how to implement them in treatment planning (Participant 9).

## Data Availability

All data are included in this study. Data are available upon request.
